# Conclusions Respecting Valvular Diseases of the Heart

**Published:** 1850-09

**Authors:** A. W. Barclay

**Affiliations:** Physician to the Chelsea, Belgrave, and Brompton Dispensary


### ARTICLE III.

*Conclusions respecting Valvular Diseases of the Heart.*---By Dr. A. W. BARCLAY, Physician to the Chelsea, Belgrave, and Brompton Dispensary.

[Dr. Barclay makes the following deductions from the analysis of a large number of cases occurring at St. George's Hospital:]

1. Liability to double valvular diseases is a consequence of rheumatic endocarditis; and this disease may attack at an early period of life, and terminate fatally at an early period. 2. A decided limit appears to be set by age to fibrinous deposit on the valves of the heart. 3. Atheromatous disease affects not by preference the mitral valve, as has been sometimes stated. 4. Though endocarditis is most commonly an acute disease, originating in connection with acute rheumatism, yet it appears that the endocardium or lining membrane of the heart is liable to become the seat either of chronic inflammation and its effects, namely, thickening and induration with corrugation of the valves, and arctation of their apertures, or of atheramatous deposition. 5. After this condition of the endocardium and valves has been insensibly established, an acute form of the disease may supervene, or hypertrophy or dilatation may ensue. 6. A large proportion, that is, nearly one-half of the fatal cases of granular kidney, is found coincident with valvular disease; but valvular disease is rare in those cases in which the kidney is large and mottled. 7. As a corollary from deduction 6, it must be admitted as probable, if not certain, that the large mottled kidney is a different disease from the granular or hard, and usually small kidney.

Points requiring farther investigation are the following :---

1. Whether the double valvular affection be the immediate, or the remote consequence of rheumatic endocarditis; and from what circumstance the disposition to renewed attack of the disease is derived? 2. Whether endocarditis ever proceeds from the enlarged and mottled state of the kidney as its sole cause; and why, among so great a number of cases connected with granular kidney, the inflammatory action was not more frequently found recent?---*Edin. Med. and Surg. Jour.*, April, 1850; p. 341.

